# An optimized DCO with modified binary-weighted DCTLs based hybrid tuning banks for an E-band DPLL

**DOI:** 10.1038/s41598-024-51510-z

**Published:** 2024-01-10

**Authors:** Lu Tang, Zichuan Yu, Yujia Lu, Zhiqi Jin, Sicong Xia, Youming Zhang, Xusheng Tang

**Affiliations:** 1 Engineering Research Centre of RF-ICs & RF-systems, Ministry of Education, Southeast University, Nanjing, 210096 China; 2 School of Cyber Science and Engineering, Southeast University, Nanjing, 211189 China

**Keywords:** Engineering, Electrical and electronic engineering

## Abstract

An optimized millimeter-wave digital controlled oscillator (DCO) in a 40-nm CMOS process is presented in this work. The coarse-tuning modules and medium-tuning modules of the DCO utilize modified binary-weighted digitally controlled transmission lines (DCTLs) to achieve a better compromise among smaller chip size, higher resonant frequency, better tuning resolution and lower phase noise. The tuning precision and die size of the medium tuning bank are improved without changing the binary coding rules by replacing the lowest-weight bit of the DCTLs with switched capacitors. In comparison with traditional DCTLs, the control bits of the coarse and medium tuning modules have been changed from 30 to 8, resulting in a 34.4% reduction in overall length (from 122$\upmu$m to 80$\upmu$m). In addition, the DCO’s fine-tuning modules are achieved using a binary-weighted switched capacitors array connected to the secondary winding of a low-coupling transformer, which enhances the DCO’s fine-tuning bank for better frequency resolution with less circuit complexity. The measured tuning range of the optimized DCO is 76-81GHz with a smaller die size of 0.12mm$^2$. This results in an outstanding figure of merit ($FoM_A$) of − 190.52dBc/Hz.

## Introduction

Digital phase-locked loop (DPLL) has been gradually adopted in millimeter-wave communication systems due to its higher level of integration, more flexible configurability, and process portability. The DCO is a key component of DPLL system. However, due to the limitations in frequency resolution, tuning range, and circuit complexity at higher millimeter-wave frequency bands, the design of DCO remains challenging in building DPLL systems.

Much research has been done over the last decade to extend the tuning range of oscillators. Tuning range extension can be achieved by increasing the number of switches and the capacitance in the inductor output legs^1^$$^,$$^2^. However, more switched capacitors will result in increased area consumption and non-linearity. Due to the additional losses introduced, the DCO may suffer from phase noise degradation and increased power dissipation to meet the start-up conditions^3^$$^,$$^4^.

Alternatively, DCTLs are used in the DCO’s tuning banks for higher Q and lower substrate loss^5^$$^,$$^6^. However, conventional DCTLs always generate a lot of parasitic capacitances due to their large die size, which will break the trade-off among the phase noise, tuning range and resonant frequency of the DCO^7^$$^,$$^8^. The frequency resolution of the DCO with a capacitive source degeneration coupling circuit topology is often affected by the transconductance of the cross-coupled transistor pair^9^. Although the switched capacitor based tuning banks can achieve a higher frequency resolution, the utilization of a significant number of capacitors also degrades the phase noise performance of the DCO^10^$$^,$$^11^. Thus, it remains a challenging task to modify the resonant tank of the millimeter-wave DCO to improve the phase noise, resonant frequency, frequency resolution and die size trade-off.

**Figure 1:**
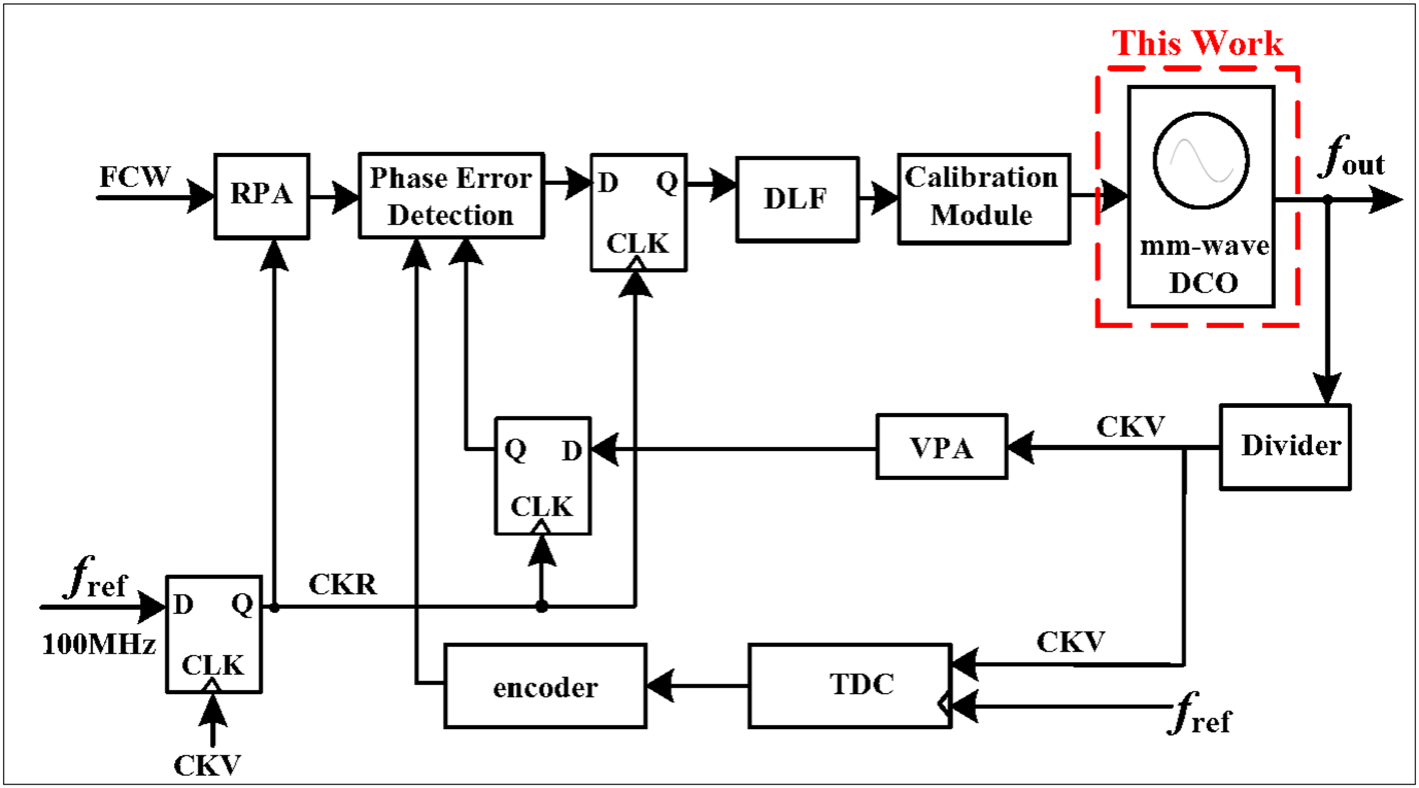
Block diagram of the E-band DPLL.

As shown in Fig. 1, The optimized DCO is integrated into an E-band DPLL. The reference phase accumulator (RPA), the variable phase accumulator (VPA), the time-to-digital converter (TDC), and the phase error detector are used for phase detection, which is similar to the phase frequency detector in a charge pump phase-locked loop. The object of quantization is the difference between VPA and TDC. The integer phase of CKV, divided clock of the DCO output frequency, is quantized by the VPA, while the difference of the fractional phase between CKV and $f_{ref}$ is quantized by the TDC. Since the generation of the fractional modulation in the DPLL loop results from the combination of other modules, further improvement of the DPLL resolution does not place significant demands on the resolution of the mm-wave DCO.

This paper presents a millimeter-wave DCO that offers a trade-off among die size, resonant frequency and phase noise. The hybrid tuning banks are described separately in subsequent section. Eventually, the experimental results demonstrate the expected characteristics of the proposed circuits.

## Design and implementation of the circuit

**Figure 2:**
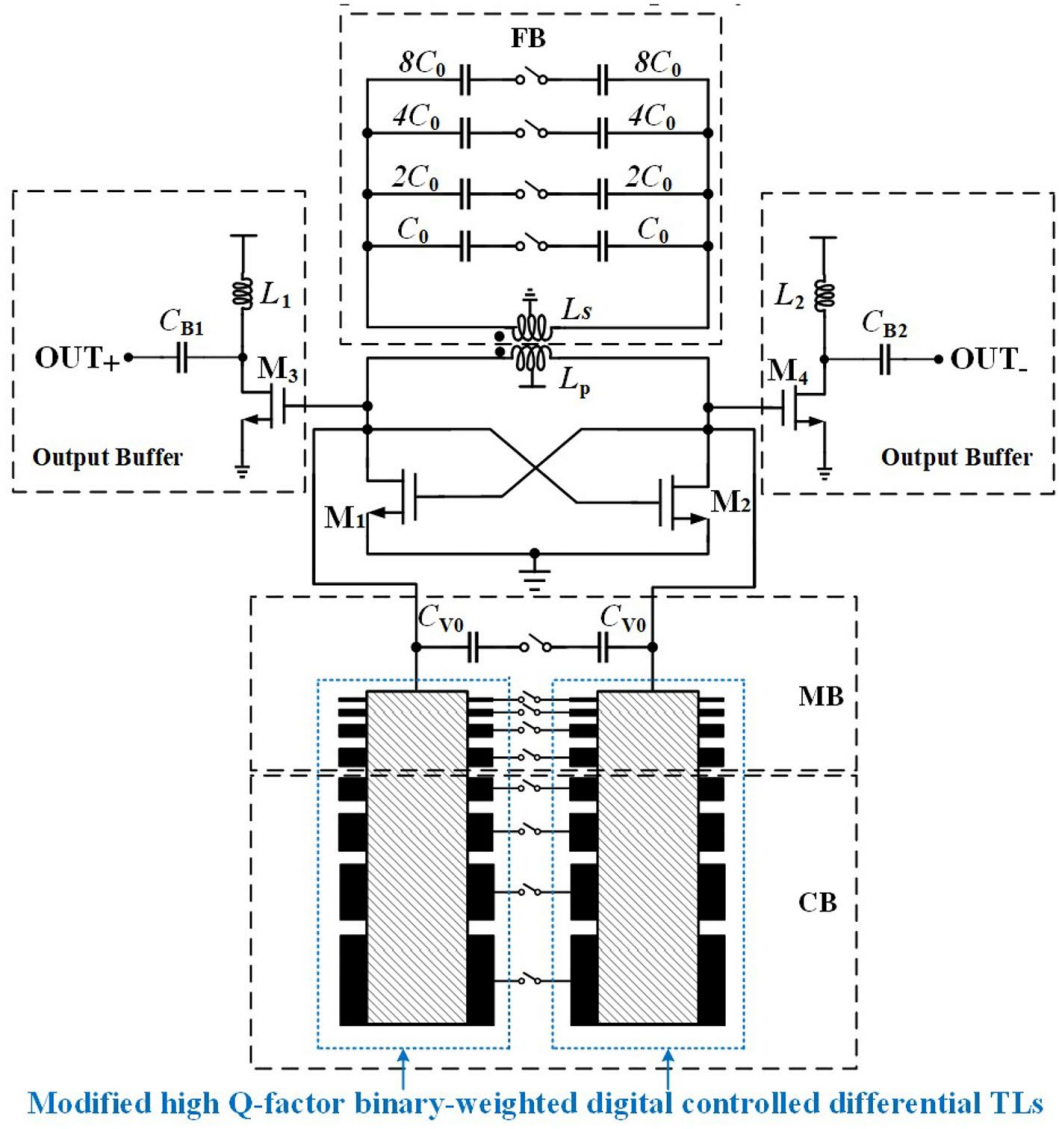
Architecture of the E-band DCO with modified binary-weighted DCTLs based hybrid tuning banks.

Figure 2 shows the circuit topology of the E-band DCO, consisting of coarse tuning bank (CB), medium tuning bank (MB) and fine tuning bank (FB). The CB is based on 4-bit modified binary-weighted DCTLs, while MB consists of 3-bit modified binary-weighted DCTLs and 1-bit switched capacitor. Additionally, the FB is composed of a 4-bit switched capacitor array connected to the secondary coil of a low-coupling transformer.

## Coarse & medium tuning modules

**Figure 3:**
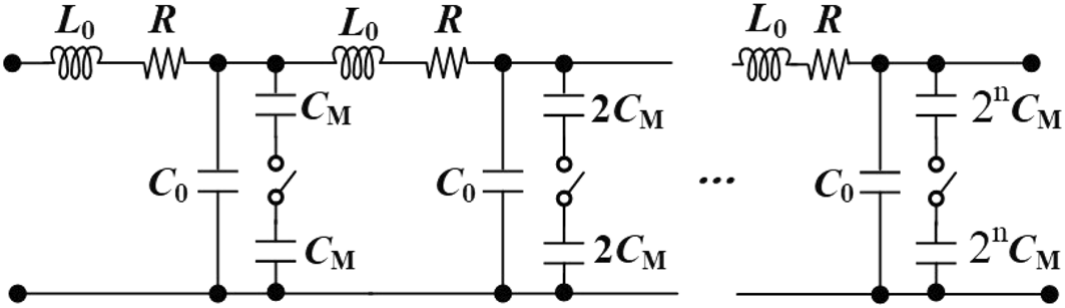
Equivalent model for binary-weighted DCTLs.

The equivalent schematic of CB and MB based on binary-weighted DCTLs is illustrated in Fig. 3. Since the proposed DCTLs change the number of capacitors connected to the tuning bank by changing the effective dielectric constant via MOS switches, this operation controls the resonant frequency at discrete frequency points according to different control codes^12^. With CM or MB connected to the circuit by the MOSFET switches, the characteristic impedance is calculated as

1 $$\begin{aligned} \begin{aligned} {Z_0} = \sqrt{{L_0}/\left( {{C_0} + {2^n}{C_M}/2} \right) } \quad \left( {0 \le n \le 3} \right) \end{aligned} \end{aligned}$$

    Where $L_0$ is the equivalent inductance of the transmission lines’ per unit length, $C_0$ is the coupling capacity between two parallel transmission lines and the capacitive coupling between the transmission lines and the substrate. $C_M$ is the Metal-Insulator-Metal capacitance between the floating strips and the transmission lines.

**Figure 4:**
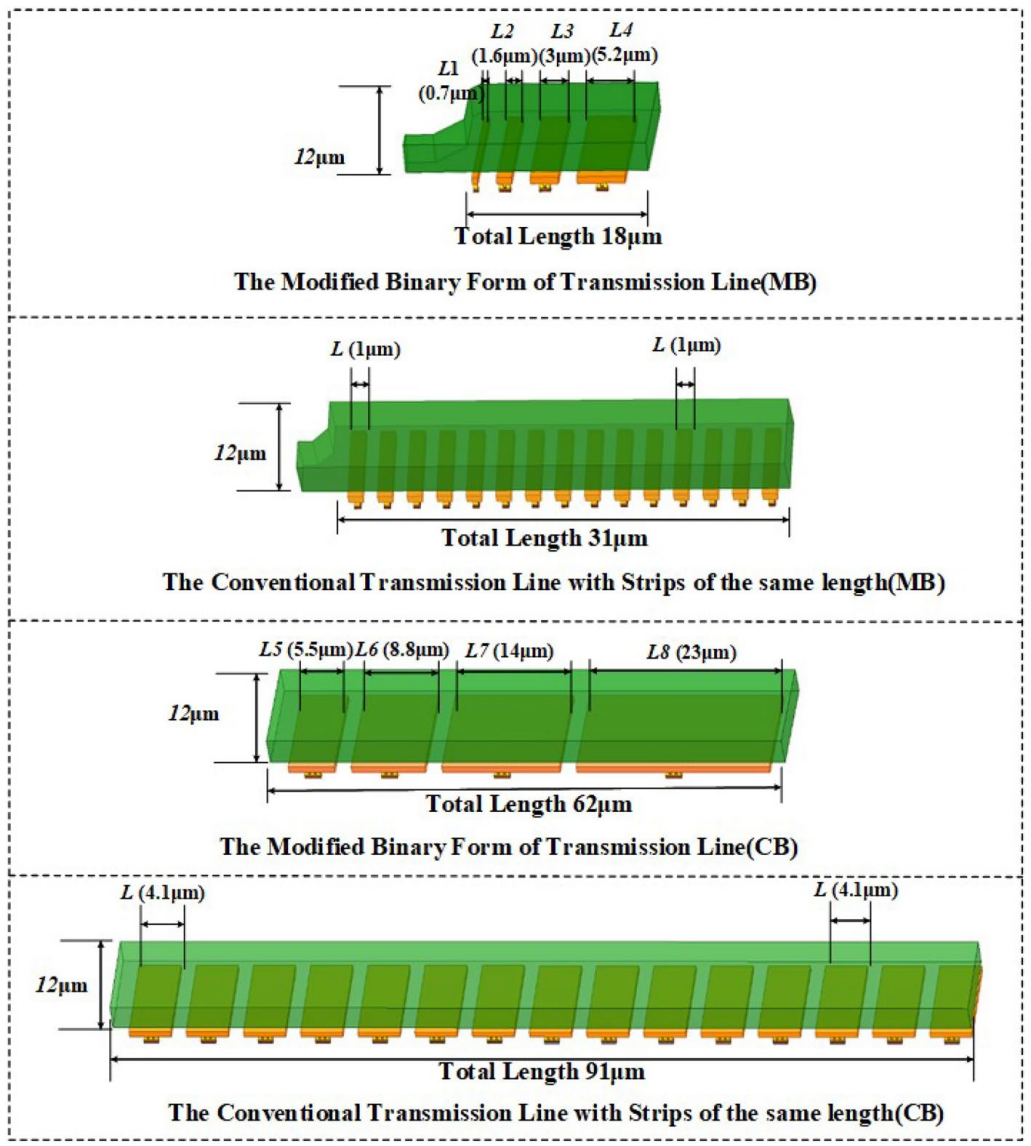
Comparison between conventional DCTLs and proposed DCTLs.

As shown in Fig.4, unlike the traditional thermometer-coded DCTLs, the floating strips used in these modified DCTLs are selectively optimized rather than strictly binary-weighted, resulting in the most linear effective dielectric constant to overcome the non-linearity problem. The CB based on the proposed DCTLs achieves a total reduction in length of 31.8% (from 91$\upmu$m and 62$\upmu$m). Similarly, the control bits of the MB (excluding the lowest weight control bit of the MB consisting of the switched capacitors) are reduced from 15 to 4, achieving a 41.9% length reduction (from 31$\upmu$m to 18$\upmu$m). The passive component loss and the fixed parasitic capacitance are reduced, while the overall Q-factor of the proposed DCTLs is increased (Q=14$\sim$ 16 @78GHz).

**Figure 5:**
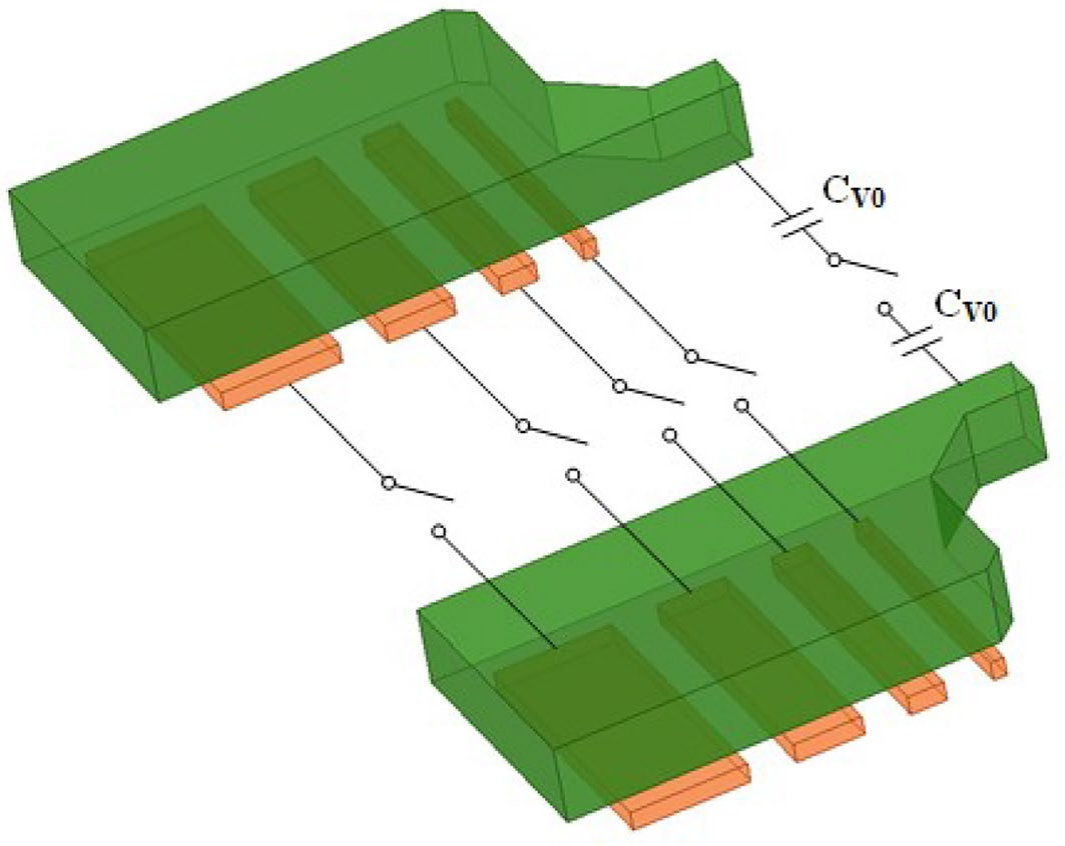
Layout of the DCTLs in MB with switching capacitors.

Figure 5 displays the structure of the hybrid modified binary-weighted DCTLs based MB. The switch capacitors are used as the lowest weight bit of the medium-tuning precision control codes, which overcomes the tuning precision limit of the DCTLs-only tuning modules without changing the binary encoding rules. At the same time, this technique reduces the die size of the MB and the impact of parasitic devices on circuit performance.

Table 1 demonstrates the comparison of the control bits between the thermometer-coded DCTLs and the proposed DCTLs applied in the CB. The tuning modules utilizing modified binary-weighted DCTLs can achieve a high resonant frequency and broad frequency tuning range with a reduced number of control bits, resulting in a more condensed chip size.

**Table 1:** Simulated $\Delta$C/bit of (a) 4-bit switch of the improved DCTLs for CB and (b) 15-bit switch of the conventional DCTLs for CB.

(a)															
4-bit Switch for the modified DCTLs	MS0	MS1	MS2	MS3											
Capacitance variation (fF/bit)	0.7	1.5	3	6											

(b)															
15-bit Switch for the conventional TLs	CS0	CS1	CS2	CS3	CS4	CS	CS6	CS7	CS8	CS9	CS10	CS11	CS12	CS13	CS14
Capacitance variation (fF/bit)	0.63	0.63	0.66	0.72	0.78	0.75	0.78	0.79	0.81	0.82	0.85	0.85	0.86	0.87	0.84

## Fine tuning module

**Figure 6:**
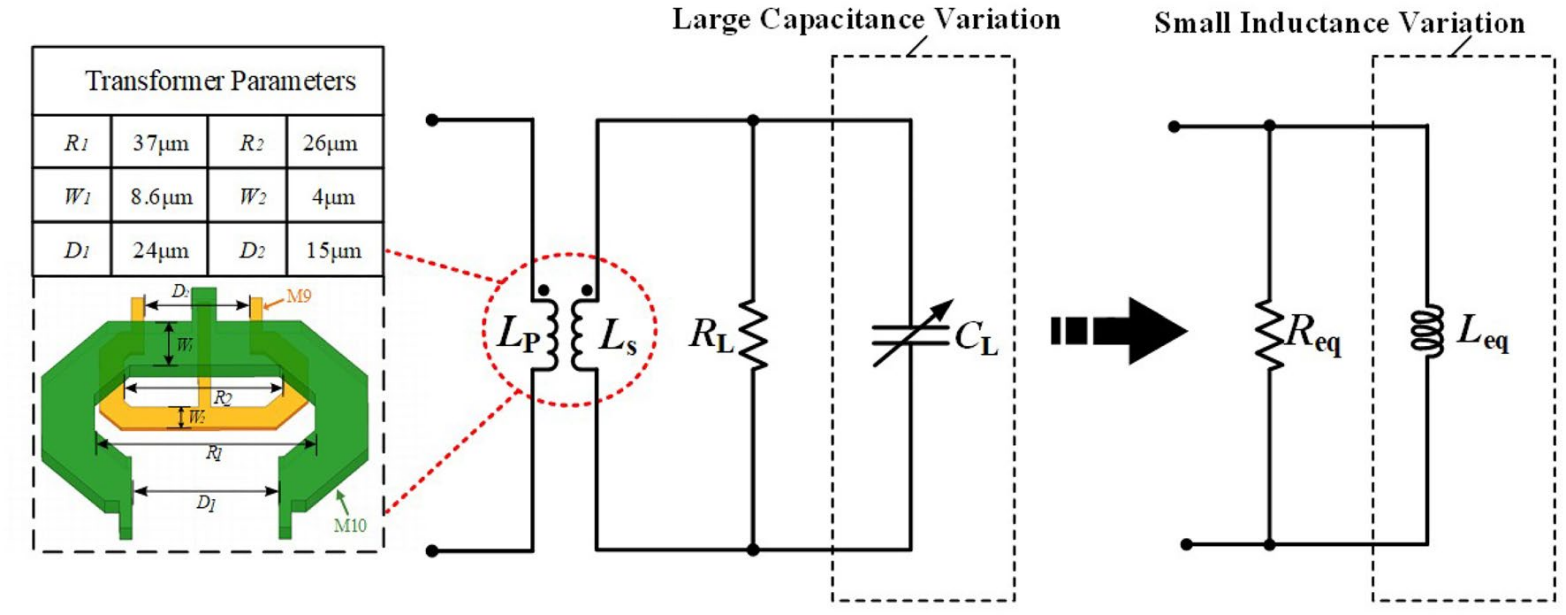
Simplified equivalent model of the FB fine tuning bank.

The simplified equivalent schematic of the FB and the low coupling coefficient transformer is shown in Fig. 6. The FB is achieved by using an array of binary capacitors connected to the secondary coil of the transformer to promote frequency resolution while reducing circuit complexity, as opposed to the typical Class F VCOs that focus primarily on enhancing the third harmonic and extending the tuning range^13–16^.

The rate of change of $L_{eq}$ with the respect to the equivalent capacitance $C_L$ of the FB can be expressed as

2 $$\begin{aligned} \begin{aligned} \frac{{\partial {L_{eq}}}}{{\partial {C_L}}} = {L_p}{L_s}{\omega ^2}{k^2} \end{aligned} \end{aligned}$$

    Where *k* is the coupling factor of the transformer. $L_{p}$ and $L_{s}$ are the respective inductances of the coils of the transformer.

**Figure 7:**
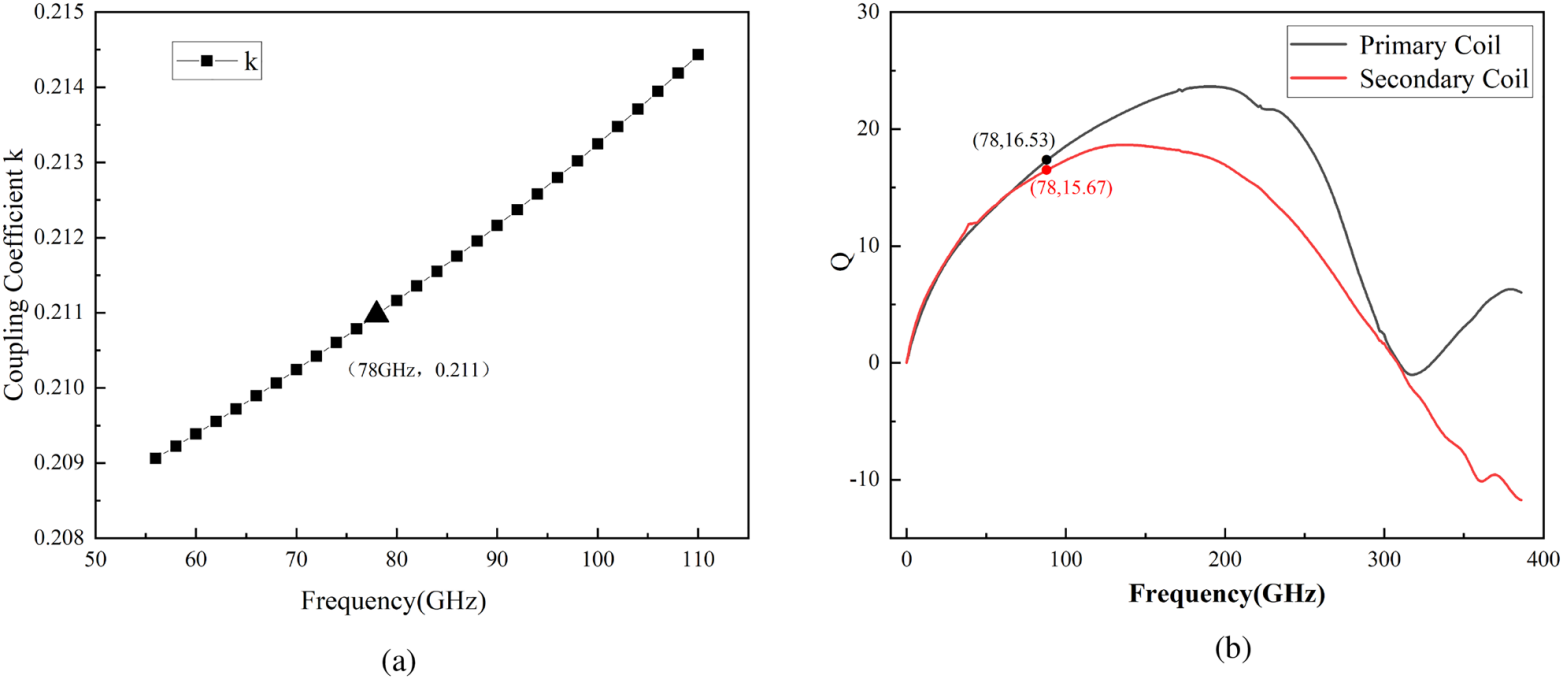
Simulated results of the transformer (**a**) coupling coefficient k (**b**) Q-factor curve.

**Figure 8:**
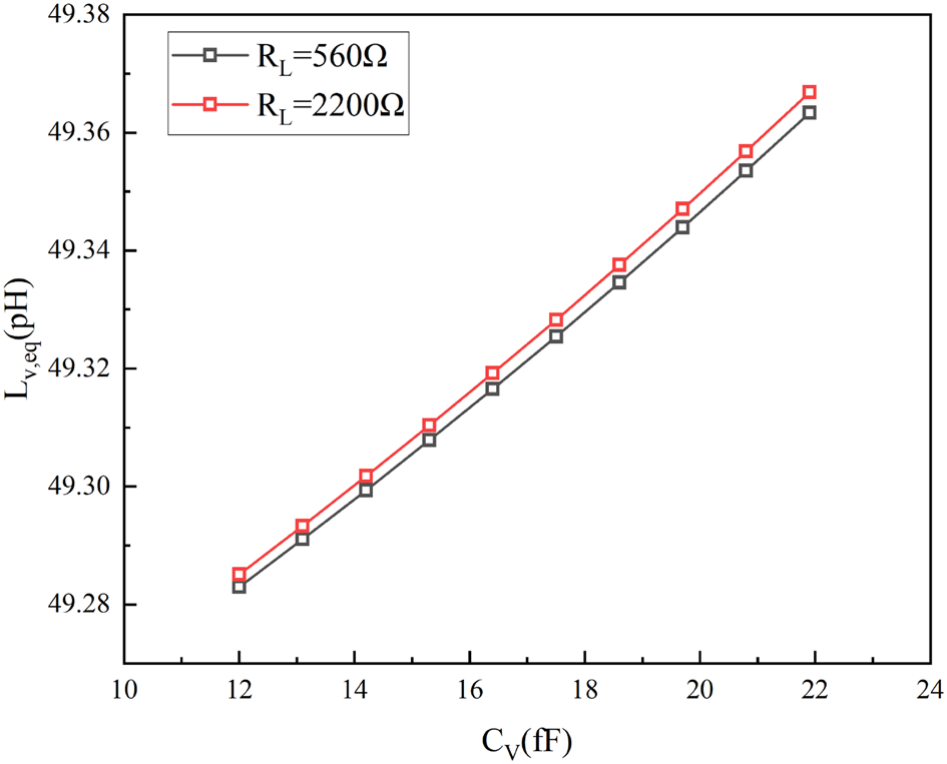
Simulated results of $L_{eq}$ for variations in $C_L$ and R.

The design of the transformer is a crucial part of the overall DCO and particular attention should be paid to the coupling coefficient and the Q-factor. The lower coupling coefficient *k* allows $L_{eq}$ to increase smoothly and linearly with the increase in $C_L$, as shown in equation (2). Figure 7 shows that the Q of the two transformer coils at the operating frequency is above 15 and the coupling factor of the transformer is below 0.25. To minimize the frequency tuning step, the coupling coefficient of the transformer *k* is set to 0.21. In addition, the detrimental effects of parasitic capacitance in the tuning bank can also be reduced by using a coupled transformer with such a small *k*^17–19^. The binary-weighted array of switched capacitors connected to the transformer’s secondary coil can be used to achieve smaller fine-tuning steps. Figure 8 shows that when the equivalent capacitance of the switched capacitor array ($C_L$) is between 12fF and 23fF and the equivalent parallel resistance ($R_L$) is between 560$\Omega$ and 2200$\Omega$, the corresponding equivalent tank inductance ($L_{eq}$) is between 49.28pH and 49.38pH. This means that a large variation in $C_L$ is converted into a small variation in the inductance of $L_{eq}$ by the transformer coupling technique.

## Measurements

**Figure 9:**
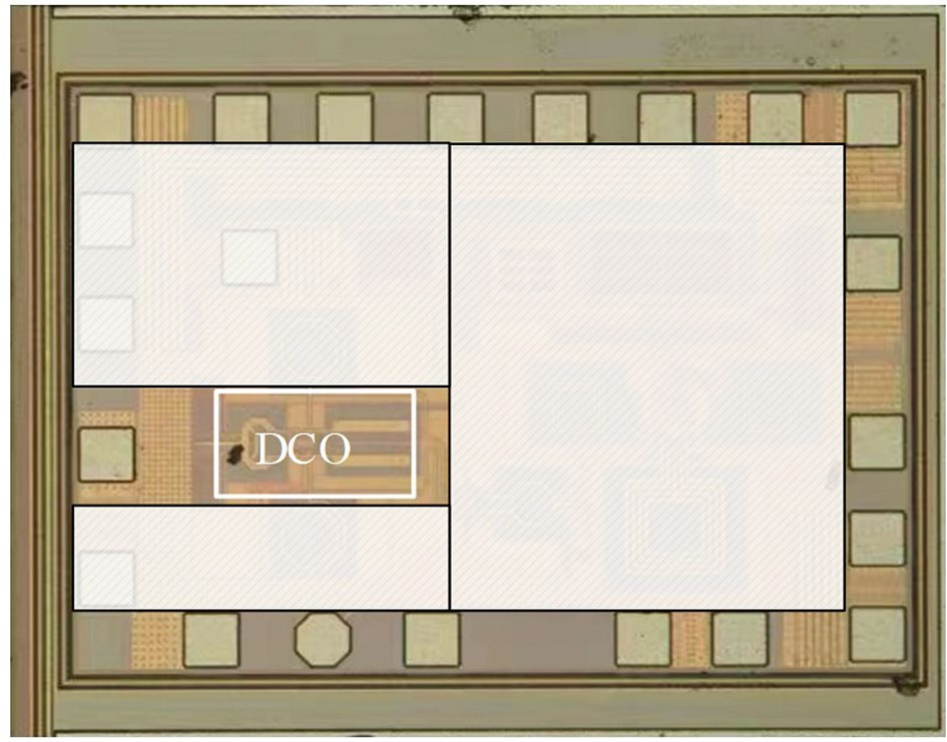
Chip micrograph of the DPLL containing the DCO.

**Figure 10:**
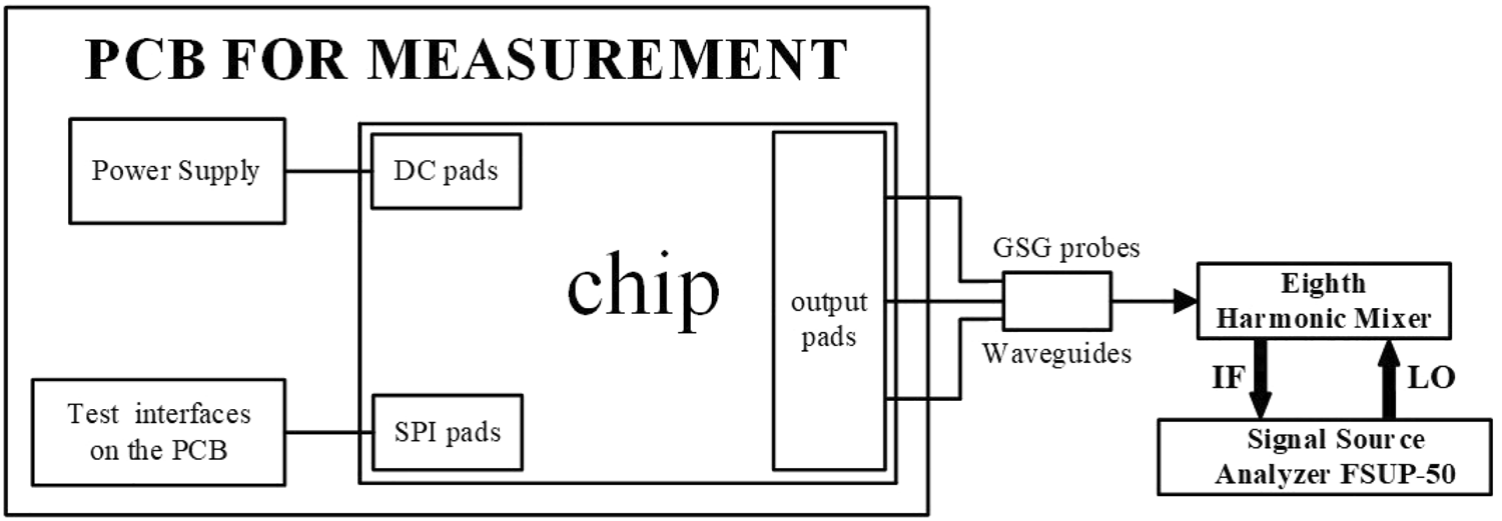
Schematic diagram of the DCO measurement setup.

The optimized DCO was integrated into an E-band DPLL and fabricated on a 40 nm CMOS process as shown in Fig. 9. As shown in Fig. 10, the performances of the DCO were measured using high frequency GSG probes, spectrum analyzer and phase noise analyzer. The die area of the DCO core is 0.48mm$\times$0.25mm.

**Figure 11:**
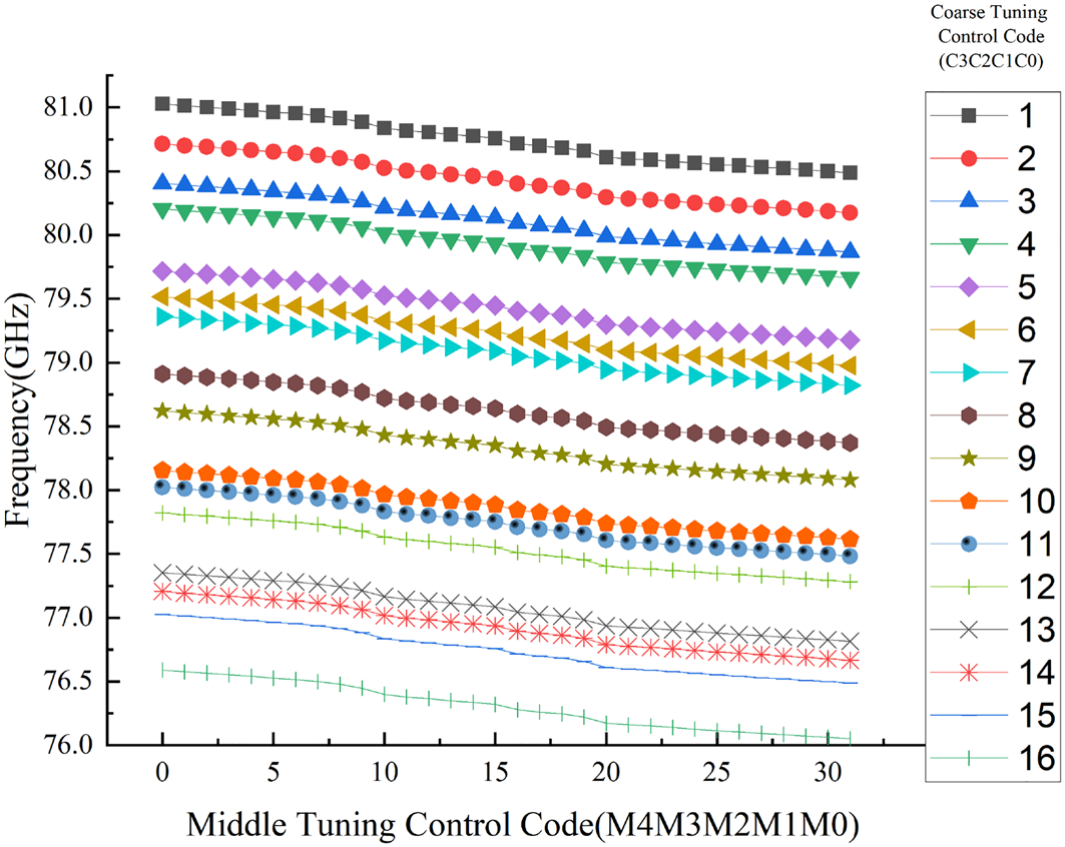
Measured medium-tuning characteristic.

**Figure 12:**
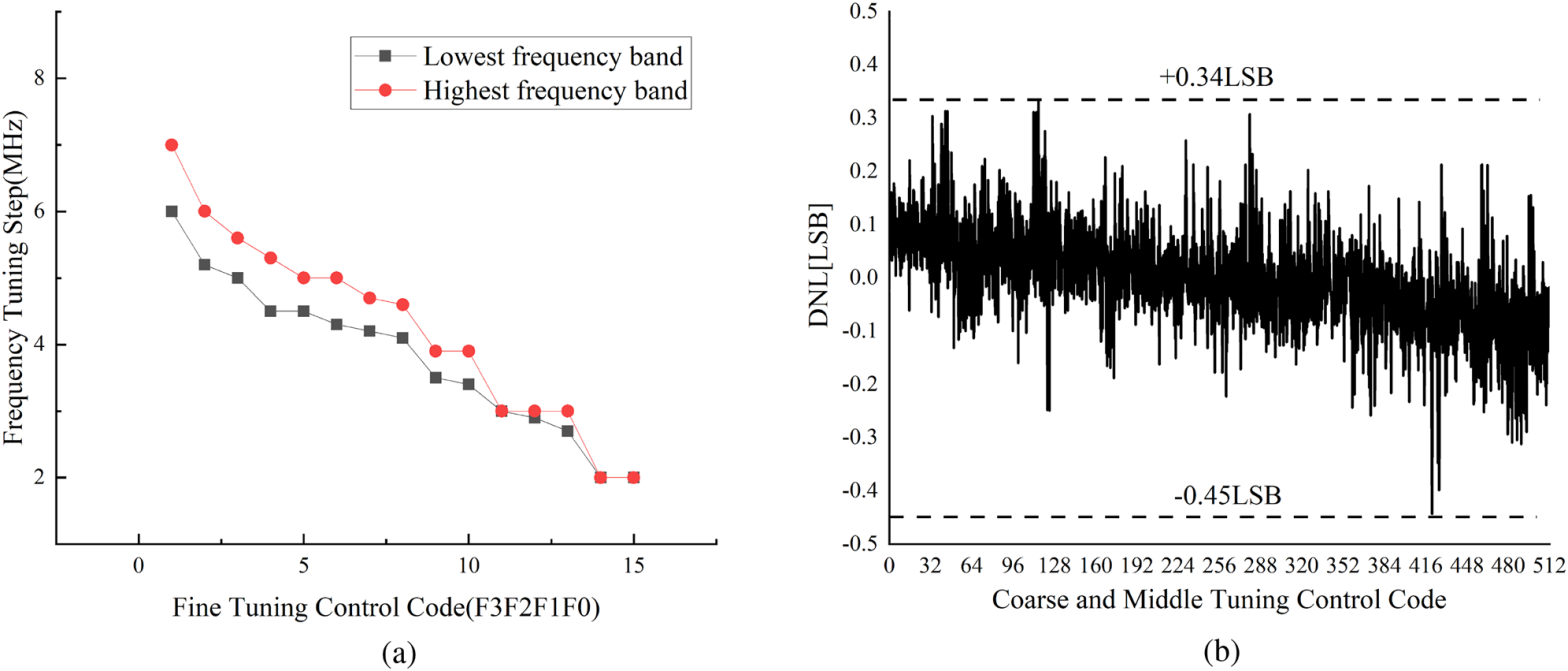
Measured (**a**) fine-tuning characteristic and (**b**) DNL of the CM and MB.

The medium-tuning curves measured under various coarse tuning codes are displayed in Fig. 11. The CB implemented by the modified binary-weighted DCTLs corresponds to 16 discrete frequency points. The average step size of the coarse-tuning can reach 297 MHz/bit, while the average step size of the medium-tuning can reach 17 MHz/bit. When the control codes of the CB are the lowest, the tuning range of the MB is 80.5-81.03GHz (530MHz), which can fully cover the maximum coarse-tuning step. Figure 12(a) shows the measured fine-tuning characteristic. If the control codes of both the MB and CB are at lowest, steps from 2 to 7 MHz can be achieved for frequency tuning. But with the control codes of the CB at 16 and the MB at 32, the frequency tuning step is from 2 and 6MHz. This means that a continuous tuning range of 76.23-76.29 GHz (60 MHz) can be achieved in the lowest frequency band, which fully covers the largest medium-tuning step size.Figure 12(b) shows the measured differential non-linearity (DNL) of the CB and MB of the DCO. A total of 8 chips were tested, of which 5 chips gave the consistent results. While the control code of the FB remains constant, the measured DNL is -0.45 LSB in the worst case among 5 chips.

**Figure 13:**
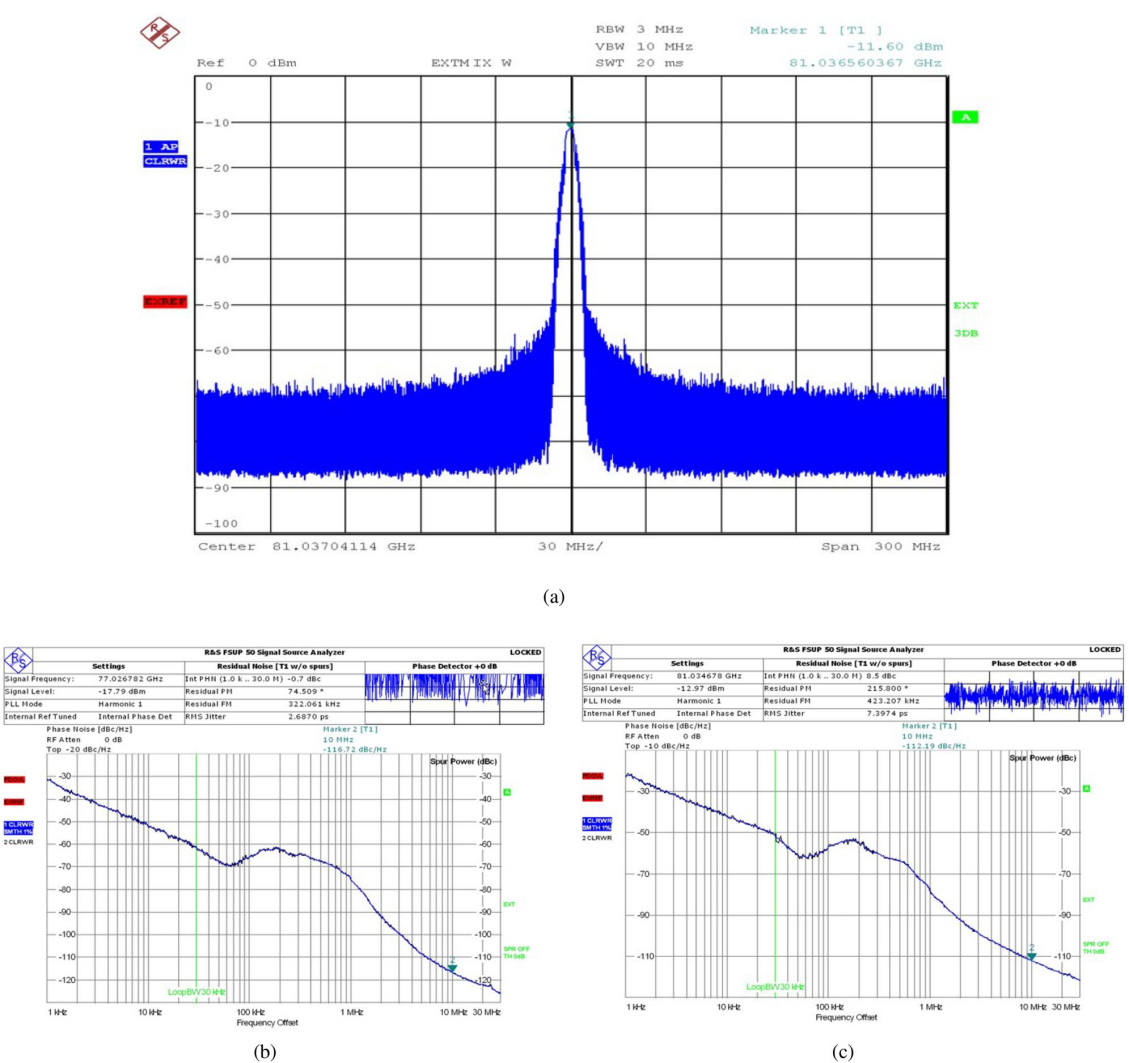
Measured results of (**a**) DCO spectrum, (**b**) DCO phase noise spectrum @ 77GHz and (**c**) DCO phase noise spectrum @ 81GHz.

Figure 13 exhibits the measured results of the DCO’s spectrum and phase noise spectrum. The DCO is tuned to oscillate at 76-81 GHz. The phase noise of the output signal at 10MHz offset is − 116.72dBc/Hz at 77GHz. The power dissipated of the DCO core is approximately 21.5mW at 0.9V supply voltage. The hump in the phase noise curve is caused by the up-conversion of the power supply noise by the DCO’s the cross-coupled amplifier, which creates multi-frequency peaks near the oscillation frequency. The DCO achieves the required metrics for the DPLL system and is able to meet the system requirements.

The proposed DCTLs result in a 34.4$\%$ reduction in the overall length of the CB and MB, while maintaining a high Q-factor (14$\sim$16 @78GHz), suggesting that it is not necessary to increase the inductor size excessively to prevent phase noise degradation of the resonant tank. As a result, the die area of the proposed DCO is reduced to some extent. Table 2 shows that the above features help to achieve an excellent balance between die size, phase noise, frequency resolution and resonant frequency, resulting in exceptional FoM and $FoM_A$^20^, especially in terms of die area.

**Table 2:** Performance comparison.

		This work	JSSC’18^13^	JSSC’19^21^	NEWCAS’20^22^	TCAS’20^23^	TMTT’20^24^
Center frequency(GHz)		78.635	94	62.35	77.8	75.45	96
Tuning ratio (%)		6.1	20.9	15.6	3.5	4.43	27.1
Phase noise(dBc/Hz)	1MHz	NA	− 103.1	− 99.6	NA	NA	− 92.5
	10MHz	− 116.72	− 124.0	− 119.1	− 114.9	− 112	NA
FoM(dBc/Hz)		− 181.31**	− 185.42**	− 180.3**	− 180.96**	− 180**	− 167.9*
FoM$_A$(dBc/Hz)		− 190.52	189.19	− 184.5	− 185.28	− 184	− 178.87
FoM$_T$(dBc/Hz)		− 177.02	− 191.82	− 184.16	− 171.84	− 172.93	− 176.56
P$_{DC}$(mW)		21.5	63.7	29.5	15	9.08	300
Die area(mm$^2$)		0.12	0.42	0.38	0.37	0.4	0.08
CMOS process		40nm	65nm	65nm	55nm	55nm	SiGe BiCMOS

*@$$\Delta$$*f=1MHz*,**@$$\Delta$$*f=10MHz*,

*FoM = PN* - 20*log* $_{10}$((*f* $_0$/$\Delta$*f*)) + 10*log* $_{10}$*(PDC/1mW)*

*FoM* $_A$= *FoM* + 10*log* $_{10}$(*Area/1mm* $^2$)

*FoM* $_T$= *FoM* -20*log* $_{10}$(*TR*[%]/10)

## Conclusion

A millimeter-wave DCO has been fabricated on a 40-nm CMOS process, featuring a trade-off among chip size, phase noise, frequency resolution, and resonant frequency. The MB and CB, which are based on binary-weighted DCTLs, and the FB, which is a binary-weighted switched capacitor array connected to the secondary winding of a low-coupling transformer, are integrated into the DCO’s resonance tank. The modified DCO reaches a high $FoM_A$ of -190.52dBc/Hz with a resonant frequency range from 76 to 81GHz, while the frequency resolution is 2MHz.

## Data Availability

The data that support the findings of this study are available from the corresponding author L.T., upon reasonable request.
